# On the origins of the mitotic shift in proliferating cell layers

**DOI:** 10.1186/1742-4682-11-26

**Published:** 2014-05-27

**Authors:** William T Gibson, Boris Y Rubinstein, Emily J Meyer, James H Veldhuis, G Wayne Brodland, Radhika Nagpal, Matthew C Gibson

**Affiliations:** 1 California Institute of Technology, 91125 Pasadena, CA, USA; 2 Stowers Institute for Medical Research, 64110 Kansas City, MO, USA; 3 Department of Civil and Environmental Engineering, University of Waterloo, N2L 3G1 Waterloo, ON, Canada; 4 School of Engineering & Applied Sciences, Harvard University, 02138 Cambridge, MA, USA; 5 Department of Anatomy and Cell Biology, Kansas University Medical Center, 66160 Kansas City, KS, USA

**Keywords:** Cell topology, Epithelial morphogenesis, Mathematical modeling, Mitosis, Developmental biology

## Abstract

**Background:**

During plant and animal development, monolayer cell sheets display a stereotyped distribution of polygonal cell shapes. In interphase cells these shapes range from quadrilaterals to decagons, with a robust average of six sides per cell. In contrast, the subset of cells in mitosis exhibits a distinct distribution with an average of seven sides. It remains unclear whether this ‘mitotic shift’ reflects a causal relationship between increased polygonal sidedness and increased division likelihood, or alternatively, a passive effect of local proliferation on cell shape.

**Methods:**

We use a combination of probabilistic analysis and mathematical modeling to predict the geometry of mitotic polygonal cells in a proliferating cell layer. To test these predictions experimentally, we use Flp-Out stochastic labeling in the *Drosophila* wing disc to induce single cell clones, and confocal imaging to quantify the polygonal topologies of these clones as a function of cellular age. For a more generic test in an idealized cell layer, we model epithelial sheet proliferation in a finite element framework, which yields a computationally robust, emergent prediction of the mitotic cell shape distribution.

**Results:**

Using both mathematical and experimental approaches, we show that the mitotic shift derives primarily from passive, non-autonomous effects of mitoses in neighboring cells on each cell’s geometry over the course of the cell cycle. Computationally, we predict that interphase cells should passively gain sides over time, such that cells at more advanced stages of the cell cycle will tend to have a larger number of neighbors than those at earlier stages. Validating this prediction, experimental analysis of randomly labeled epithelial cells in the *Drosophila* wing disc demonstrates that labeled cells exhibit an age-dependent increase in polygonal sidedness. Reinforcing these data, finite element simulations of epithelial sheet proliferation demonstrate in a generic framework that passive side-gaining is sufficient to generate a mitotic shift.

**Conclusions:**

Taken together, our results strongly suggest that the mitotic shift reflects a time-dependent accumulation of shared cellular interfaces over the course of the cell cycle. These results uncover fundamental constraints on the relationship between cell shape and cell division that should be general in adherent, polarized cell layers.

## Introduction

**Figure 1:**
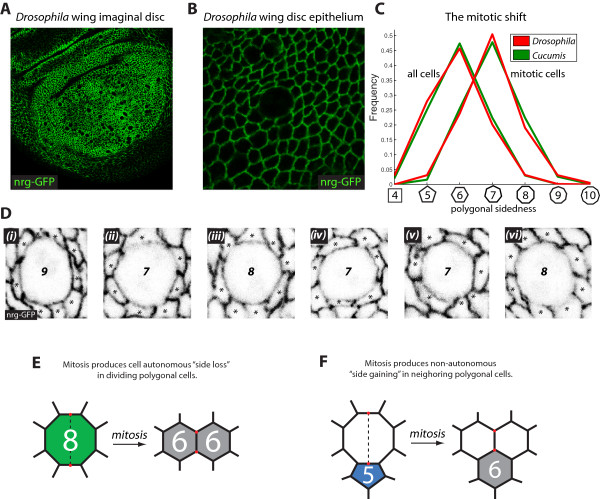
**Introduction to the mitotic shift. (A)** The *Drosophila* wing imaginal disc. Neuroglian-GFP (*green*) marks the septate junctions. **(B)** The *Drosophila* wing disc epithelium. The rounded cell (*center*) is undergoing mitosis. **(C)** The mitotic shift in *Drosophila* (*red*) and *Cucumis* (*green*) [9,24]. The overall distribution of cellular shapes has a hexagonal mean (*red* and *green*). By contrast, the mitotic cell shape distribution (*red* and *green*) is shifted to have a heptagonal mean in both organisms. Hence, one distribution is approximately a shifted version of the other. Sample sizes for both organisms are given under the heading “Sample sizes for overall and mitotic cell shape distributions” in the Methods section. **(D)** Representative mitotic cells (as detected based on cell rounding) in the *Drosophila* wing imaginal disc. Mitotic cells show an enrichment in cell-cell contacts. Stars (*black*) mark neighboring cells; labeled cells’ polygonal topologies are designated in black. **(E-F)** An overview of mitotically induced topological transformations during epithelial proliferation. **(E)** An illustration of autonomous “side loss” during polygonal cell division. The octagonal cell (*green*) gives rise to two hexagonal daughters (*grey*). On average, a mitotic *N*-sided cell will give rise to daughters having (*N* + 4)/2 sides, making side loss a general trend except for rare polygonal cells in which *N* = 3 or *N* = 4. Note the creation of a set of new tri-cellular junctions (*red*), which are formed at either end of the cleavage plane (*black*), which is depicted as a dashed line. **(F)** Non-autonomous “side gaining” due to neighbor cell mitoses. In this example, a pentagonal cell (*blue*), gains one neighbor to become a hexagonal cell (*grey*). In general, during polygonal cell division, exactly two neighboring cells adjacent to the newly-formed tri-cellular junctions (*red*) will effectively gain one neighbor each.

Cellular structures are ubiquitous in natural systems, from the crack patterns of ceramic glazes and colloidal suspensions [1-4], to the bubble structures of a coarsening foam [5-8], to the complex tissues of living organisms [9-13] (Figures 1A-B). The subset of two-dimensional (planar) cellular structures constitutes an analytically tractable paradigm for uncovering the fundamental geometrical and mathematical constraints imposed by cell packing that govern cell shape in a contiguous tissue [5,14,15]. In a biological context, combined with physical forces [16-18], these constraints illuminate a non-genetic basis for biological form, and restrict the space of possible tissue architectures. Packing considerations impose powerful constraints on diverse features of cellular geometry, including area [9,14], topological neighbor correlations [15], distributions of the number of cellular neighbors [5,10,12,19], and the average polygonal shape of a given cell (which is hexagonal [20]), among others [21]. Beginning with D’Arcy Thompson and Frederic Lewis, it was appreciated in the early 20^th^ century that such constraints might influence or correlate with important biological variables pertaining to the growth of tissues during development [9,22]. More recent work has suggested that packing constraints are likely to be involved in the coordination between proliferative growth and morphogenesis, as these processes are intrinsically linked in growing cell layers [12,17,23-27].

In a network of adherent cellular polygons, cell shape emerges both from autonomous and from non-autonomous effects of cell division. Mitosis alters cell geometry cell-autonomously by reducing the number of neighbors of a dividing cell (i.e., an octagon may divide into a pair of hexagons, resulting in “side loss”; Figure 1E). Simultaneously, mitosis acts cell non-autonomously by generating new neighbor interfaces for cells adjacent to the recent site of division, which results in “side gaining” (Figure 1F). Numerous theoretical and simulation studies, in combination with live-imaging experiments and clonal analysis in *Drosophila*, suggest that side gaining and side loss drive epithelial cell shape emergence via cell division and cell sorting, with cell division being the dominant influence [5,10,12,19,24,27,28].

Notably, despite the broad range of theoretically possible cell shape distributions [29-31], cell layers in many plant and animal species nevertheless converge on a conserved equilibrium distribution having approximately 25% pentagons, 45% hexagons, and 20% heptagons [9,12,32]. The form of the distribution is likely constrained entropically [5]. Intriguingly, for both *Drosophila* (a representative animal model system) and *Cucumis* (a representative plant model system), the form of the mitotic cell shape distribution is nearly identical to the overall distribution, with the critical difference being that it is shifted by a single polygon class to have a heptagonal mean, in contrast to the hexagonal mean characteristic of the overall distribution (shown in Figures 1C-D). Hence, despite the independent evolutionary origins of plant and animal multicellularity [33], it appears that both are governed by fundamentally similar topological constraints.

Although the existence of the single-integer mitotic shift may imply a fundamental correlation between polygon class and division likelihood in proliferating cell layers, its cellular basis remains unclear. The chicken-egg nature of the problem centers on how to interpret the shift in terms of the mitotic cell cycle. For instance, one possibility is that increased cell sidedness promotes mitotic entry, although there is no functional evidence to support this view [28,34]. An alternative interpretation is that over time, interphase cells simply gain sides as a passive consequence of adjacent mitotic events [5,12,24,32,35]. Under steady-state assumptions, for instance, a shifted (heptagonal) mean and mitotic distribution can be predicted algebraically [5,35]. Hence, rather than indicating active cell-cycle regulation, the mitotic shift could reflect an emergent interaction between cell packing and heterogeneous proliferation. Here, in order to resolve this problem, we develop a novel mathematical framework to explicitly define the implications of non-autonomous side gaining for the mitotic cell shape distribution in cellular monolayers featuring tight cell adhesion and negligible rearrangements. Our computations predict that interphase cells should passively gain sides over time, such that cells that are more advanced in the cell cycle will tend to have a larger number of neighbors. This inference is borne out by experimental analysis of proliferating *Drosophila* epithelial cells as well as by finite element simulations of proliferating epithelia. We argue that the mitotic shift is likely to be a widespread geometrical feature of adherent, proliferating cellular monolayers in plants and animals.

## Results

### Defining the logical relationship between polygon class and mitotic entry

The existence of the mitotic shift implies that within cell sheets, the probability of cell division *F(N)* must correlate with polygon class *N*. To show this, assume there is no such correlation, meaning that all polygon classes undergo mitosis with the same probability per unit time. Under these conditions, at steady-state, the fraction of *N*-sided polygonal mitotic cells would be identical to the fraction of *N*-sided polygonal cells overall (Additional file 1: Figure S1), contradicting the existence of the nearly identical single integer mitotic shifts observed in plant and animal cell layers [9,12,28]. Therefore, irrespective of the underlying mechanism, the mitotic shift implies that division probability and hence cell cycle state correlates with polygon class.

Similar reasoning leads to a second insight, which is that for tissues exhibiting a mitotic shift, cells cannot have perfectly synchronized cycles. For the case of perfectly synchronized cell cycles, all cells in the cell layer would divide simultaneously at each round of division. To show that this synchronized scenario cannot exist simultaneously with a mitotic shift, assume perfect mitotic synchrony in a proliferating cell layer (ie, a situation in which all cells divide simultaneously). Under these conditions, the distribution of mitotic and non-mitotic cells would be identical at steady-state, contradicting the existence of the shift. As a consequence of asynchronous proliferation, a time delay will necessarily exist between the divisions of neighboring cells. As a result, the average interphase cell will tend to gain additional cell-cell contacts from its apposed mitotic neighbors over the course of the cell cycle. Based on this logic, the intuitive expectation is that within cell sheets, asynchronous division will result in a positive correlation between polygon class and cell cycle state. Indeed, empirical data from previous studies has suggested that, on average, cells having more sides are more likely to undergo mitosis (according to multiple metrics, including metaphase marker staining, cell rounding, and cytokinesis [12,24]).

### A positive correlation between polygon class and cell cycle state is the default expectation, and a trend across diverse organisms

In order to formalize the above reasoning, we can write the probability *F(N)* that an *N*-sided cell will undergo mitosis per time step in terms of the following three quantities: (1) the probability that a cell undergoing mitosis has *N* neighbors, *P(N|D),* which is equivalently the polygonal cell shape distribution for dividing cells; (2) the average fraction of cells in the epithelium undergoing mitosis, *P(D)*; and (3) the probability *P(N)* that a randomly selected cell in the epithelium has *N* neighbors. These quantities are related in the following manner:

(1) $$F\left(N\right)=\frac{P\left(N|D\right)P\left(D\right)}{P\left(N\right)},$$

Assuming the case of a perfect integer mitotic shift (which is a close approximation empirically), the mitotic cell shape distribution *P(N|D)* is equal to *P(N-1)*. We find,

(2) $$F\left(N\right)=\frac{P\left(N-1\right)P\left(D\right)}{P\left(N\right)},$$

and hence,

(3) $$P\left(N\right)=\frac{P\left(D\right)}{F\left(N\right)}P\left(N-1\right).$$

Equation (3) implies that the distribution *P(N)* achieves its maximum when the function *F(N)* crosses the average division rate *P(D)* from below. The fact that *P(N)* is uni-modal empirically indicates that *F(N)* crosses the value *P(D)* exactly once, meaning that all values on the right side of the crossing point stay above *P(D)*, and all values on the left side of it stay below. Therefore, higher-order polygon classes tend to have a greater division probability than lower-order polygon classes. The uni-modal character of the shape distribution *P(N)* is observed in diverse plant and animal species (and also in simulations), suggesting that this reasoning may be general [9,12,28-30,32,36]. Consistent with the above analysis, when *F*(*N*) is assumed to have an exponential form (which is approximately true for the *Drosophila* wing disc epithelium [24] and for the epidermis of *Cucumis *[19]), equation (3) implies that the distribution of polygonal cell shapes has the following form (with parameters *p*_*1*_ and *p*_*2*_; $p_{2}\approx \frac{1}{p_{1}^{2.5}}$ for 〈*N*〉 ≈ 6): $P\left(N\right)=\frac{1}{\sum_{i=4}^{\infty }\left[p_{1}{\left(\frac{i-3}{2}\right)}^{\left(i-4\right)}{p_{2}}^{\left(i-4\right)}\right]}p_{1}{\left(\frac{N-3}{2}\right)}^{\left(N-4\right)}{p_{2}}^{\left(N-4\right)}$, which is uni-modal.

While the above result confirms that higher order polygons are more likely to divide, it does not show that division probability should increase with every individual polygon class *N*, which would require the following condition based on equation (1):

(4) $$\frac{P\left(N+1\right)}{P\left(N\right)}<\frac{P\left(N\right)}{P\left(N-1\right)}\forall N.$$

Given that *P(N)* is uni-modal, equation (4) would constrain its overall shape. Specifically, for values of *N <* 6, the fold change increase of *P(N)* must decline for each *N*, and thus the fold-change is bounded from above. Conversely, for values of *N >* 6, the fold change decrease of *P(N)* must rise for each *N* such that the fold-change is bounded from below. While there is no theoretical basis for assuming this constraint applies to all tissues, the relationship in equation (4) does hold in diverse organisms, including *Drosophila* and *Cucumis* (see Table 1). Therefore, on an empirical basis, it looks to be a general trend.

**Table 1:** **Diverse organisms obey the constraint specified in equation (**4**)**

**Species name**	**5-sided:4-sided ratio**	**6-sided:5-sided ratio**	**7-sided:6-sided ratio**	**8-sided:7-sided ratio**	**9-sided:8-sided ratio**
*Drosophila*	9.469	1.639	0.440	0.158	0.044
*Cucumis*	12.550	1.888	0.473	0.134	0.033
*Xenopus*	7.625	1.479	0.424	0.272	0.154
*Hydra*	9.938	1.748	0.450	0.184	0.044
*Allium*	7.550	1.364	0.439	0.260	0.319
*Euonymus*	9.666	1.379	0.550	0.300	0.000
*Dryopteris*	6.500	1.635	0.482	0.244	0.400
*Anacharis* (leaf, abaxial)	9.600	2.479	0.1681	0.350	0
*Anacharis* (leaf, adaxial)	7.286	2.235	0.2281	0.077	0
*Anacharis* (bud)	5.364	1.525	0.444	0.000	Undefined

For each distribution, we computed the following five ratios of cell shape frequencies: (1) pentagonal to quadrilateral frequencies, (2) hexagonal to pentagonal, (3) heptagonal to hexagonal, (4) octagonal to heptagonal, and (5) nonagonal to octagonal frequencies. Equation (4) predicts that these ratios should decrease across each row. Note that diverse organisms satisfy this condition, which corresponds to equation (4) in the text. Sample sizes for each organism are given under the heading “Sample sizes and polygonal counts by organism” in the Methods section.

### Modeling the emergence of the mitotic shift in terms of cellular age

Building on the logic of the previous two sections, we next developed a mathematical model for the mitotic shift. Previous studies have considered the emergence of polygonal cell shape in proliferating epithelia, but none have formulated an analytical approach to describe the side-gaining process as it relates to cell cycle state [5,10,19]. Here, based on a set of simple topological rules, we present a mathematical description for the side gaining process in proliferating epithelia. We first make the following three assumptions, which increase analytical tractability but are not expected to substantially alter our results:

(1) The distribution of neighboring cells surrounding dividing cells is approximately the same as the overall polygonal distribution of cells, *P(N)*, which is at steady-state. This is approximately true empirically in the *Drosophila* wing disc [24].

(2) Cell rearrangement can be neglected for the case of a single round of division. Multiple lines of evidence are consistent with this view [9,12,24], which simplifies analytical treatment, although it is straightforward to simulate a scenario in which rearrangements are present. Moreover, the results of the model can be directly compared with clone experiments in *Drosophila* to test for any potential role of rearrangement.

(3) For analysis (including mathematical summations), we assume that all cells in the epithelium have between 4 and 9 sides, which is an empirical fact in both *Drosophila* and *Cucumis*, save for very rare 10-sided cells, which have negligible frequency.

We define the conditional probability *Q*_*w*_(*m*) that a dividing *w*-sided cell orients its cleavage plane so as to cleave its common interface with an *m*-sided neighbor. The computations below use the empirically measured values of a mean-field function *Q(m)*, which is an average of the function *Q*_*w*_*(m)* over all possible *w* values [24]. For comparison with a completely random cleavage plane, on average (denoted by angular brackets), the system has a probability $\frac{2}{\langle w\rangle }$ of cleaving a common interface with an arbitrarily selected *m*-sided neighbor of a mitotic cell.

### The side-gaining process as a binary tree

The algorithm to compute the mitotic polygonal cell shape distribution is written exclusively in terms of side-gaining events. Side-gaining is a direct consequence of neighbor cell mitosis, wherein the mitotic neighbor cleaves its common interface with the cell in question, thereby creating two edges where only one existed previously, and hence increasing the recipient cell’s polygon class by a single edge. Note that side gaining only occurs when the mitotic neighbor’s cleavage plane orients in a given cell’s direction; otherwise no such common interface is cleaved, and the cell’s polygon class remains unchanged. Side gaining is therefore a binary event; for each neighboring cell division, a cell either gains a single side or it does not.

For analysis, we assume that mitotic events occur stochastically. Using this approach, after *k* neighbor cell divisions, a polygonal cell can gain at minimum zero sides (if none of the cleavage planes point in its direction), and at most *k* sides (if all of the cleavage planes point in its direction). Side-gaining events are assumed to be independent.

**Figure 2:**
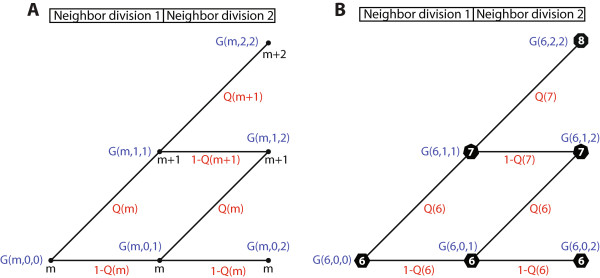
**A binary tree representation of the side-gaining process. (A)** The function *G*(*m*,*n*,*V*) represents the probability that an *m*-sided cell gains *n* sides after *V* of its immediate neighbors have divided. Side-gaining is a binary event which occurs when a neighboring cell’s cleavage plane impinges on a common interface. For each neighboring division, either one or zero sides is gained. *Q*(*m*) is the probability that an *m*-sided cell gains a side due to a single neighboring division. On the binary tree, horizontal paths represent a failure to gain a side, which occurs with probability 1-*Q*(*m*). Elevated paths represent side-gaining events. Note that to compute G(*m*,*n*,*V*), multiple paths representing different stochastic trajectories must be summed. **(B)** A more concrete representation of the side-gaining process, which here depicts the different cell shape trajectories for a hexagon, and its potential transitions to a heptagonal or octagonal state due to side-gaining.

In order to approximately compute the subset of neighbors that divide in the orientation of the cell in question, we consider the probability *Q(m)* that an *m*-sided cell gains a new edge due to the mitosis of a neighboring cell. *Q(m)* negatively correlates with the polygon class, *m *[24]. In terms of *Q(m)*, we can compute the probability *G(m,k,V)* that an *m*-sided cell gains *k* sides after *V* of its neighbor cells have divided (see Figures 2A-B). For example, the probability *G(m,*0*,V)* that the *m*-sided cell gains zero sides after *V* divisions is the following:

(5) $$G\left(m,0,V\right)={\left(1-Q\left(m\right)\right)}^{V}.$$

This is simply the probability of not gaining a side, 1-*Q(m)*, *V* times in a row. The opposite situation occurs when every neighbor cell division results in a side-gaining event. For this case:

(6) $$G\left(m,k,k\right)=\prod_{r=0}^{k-1}Q\left(m+r\right),$$

where *r* is an index, and *G(m,k,k)* is the probability of gaining *k* sides after *k* divisions. For instance, the probability that a hexagon gains two sides after two neighbor cell divisions is just the probability *Q*([6]) that a hexagon gains one side due to a neighboring division to become a heptagon, times the probability *Q*([7]) that the newly-formed heptagon gains one side to become an octagon.

Visually, the function *G(m,k,V)* can be represented as a binary tree (Figure 2A-B). Figure 2A illustrates how multiple stochastic trajectories can lead to the same eventual side-gaining outcome. For instance, the chance that a hexagon gains zero, one, or two sides after two neighboring divisions is illustrated graphically in Figure 2B. In order to compute the chance of reaching a particular polygon class, the paths leading to that potential outcome must be added together. For instance, there is only one path leading to a hexagonal fate (Figure 2B, bottom line). By contrast, the heptagonal fate has two paths impinging on it, which must be summed to determine the chance of the hexagon transitioning to a heptagon.

Algebraically, the probability *G(m,k,V)* that an *m*-sided polygon gains *k* sides after *V* neighbor cell divisions can be computed in terms of the following recursion relation:

(7) $$\;G\left(m,k,V\right)=G\left(m,k,V-1\right)\left(1-Q\left(m+k\right)\right)+G\left(m,k-1,V-1\right)Q\left(m+k-1\right).$$

The recursion relation has two terms because there are two ways to reach *G(m,k,V)* from the previous division step *V*-1. One way is to have gained *k* sides already after *V*-1 divisions, and then to gain no sides on the *V*^th^ division. This is equivalent to following a horizontal path on the binary tree (Figure 2A). The other way is to have gained *k*-1 sides after *V*-1 divisions, and to gain the *k*^th^ side on the *V*^th^ division. This corresponds to taking one of the inclined paths on the binary tree. In this Markovian framework, it is then straightforward to compute the likelihood of each possible trajectory for the side-gaining dynamics of an *m*-sided cell.

### The stochastic dynamics of neighbor division events

Having defined the likelihood with which an *m*-sided cell gains sides due to neighboring division events, we next determined the expected number of dividing neighbors *J*_*m*_ for a polygonal cell having *m* neighbors. In particular, we computed the average number of neighbor cell divisions expected to occur prior to the division of the central *m*-sided cell. For analysis, we modeled proliferation as a Poisson process. Under the Poisson model of neighbor division events, for a given time window *L*, the probability density describing the number of times *q* that a neighboring *k*-cell will divide is the following:

(8) $$p_{k}\left(q\right)=\frac{e^{-\lambda_{k}L}{\left(\lambda_{k}L\right)}^{q}}{q!}$$

where *λ*_*k*_ is the rate parameter for a *k*-cell. For a given pair of neighboring cells, with *m* and *k* sides, respectively, the probability that the *k*-cell will divide first is the following:

(9) $$p\left(k=\mathrm{first}\right)=\frac{\lambda_{k}}{\lambda_{m}+\lambda_{k}}$$

When the *k*-cell divides first, we can set the time scale $L_{m}^{k}$ over which to compute the number of divisions of the *k*-cell prior to the *m*-cell. That is:

(10) $$L_{m}^{k}=\frac{1}{\lambda_{m}}$$

which is the average waiting time until the *m*-cell divides.

To summarize, when a *k*-sided cell neighbors an *m*-sided cell, for the subset of the times when the *k*-sided cell is expected to divide first, the distribution of the number of times *q* that the *k*-cell will divide is the following:

(11) $$p_{k}^{m}\left(q\right)=\frac{{e^{-}}^{\left(\frac{\lambda_{k}}{\lambda_{m}}\right)}{\left(\frac{\lambda_{k}}{\lambda_{m}}\right)}^{q}}{q!}$$

We can scale the above distribution by the probability that the *k*-cell divides first:

(12) $$p_{k,\mathrm{scaled}}^{m}\left(q\right)=\frac{\lambda_{k}}{\lambda_{k}+\lambda_{m}}\frac{e^{-\left(\frac{\lambda_{k}}{\lambda_{m}}\right)}{\left(\frac{\lambda_{k}}{\lambda_{m}}\right)}^{q}}{q!}$$

The distribution of *J*_*m*_ values is therefore the above expression summed over all of the *m* neighbors, and averaging over the probability of each possible type of polygonal neighbor. Hence, *p*(*J*_*m*_) is a weighted sum of *m* independent and identically distributed Poisson random variables, which reduces to the following:

(13) $$p\left(J_{m}\right)=\sum_{k=4}^{9}P\left(k\right)\frac{\lambda_{k}}{\lambda_{k}+\lambda_{m}}\frac{e^{-m\left(\frac{\lambda_{k}}{\lambda_{m}}\right)}{\left(m\frac{\lambda_{k}}{\lambda_{m}}\right)}^{q}}{q!}$$

The mean field estimate for the average value of *J*_*m*_ is the following:

(14) $$\langle J_{m}\rangle \approx m\sum_{k=4}^{9}P\left(k\right)\frac{\lambda_{k}}{\lambda_{k}+\lambda_{m}}\left(\frac{\lambda_{k}}{\lambda_{m}}\right)$$

As *λ*_*k*_ ∝ *F*(*k*), we can re-write equation (14) in the following manner:

(15) $$\langle J_{m}\rangle \approx m\sum_{k=4}^{9}P\left(k\right)\frac{F\left(k\right)}{F\left(k\right)+F\left(m\right)}\left(\frac{F\left(k\right)}{F\left(m\right)}\right)$$

When *F(k)* is an exponential function with exponential constant “*a*”, this estimate becomes the following:

(15b) Jm≈m∑k=49Pkea2k-meak+eam

We denote the fractional part of 〈*J*_*m*_〉 as {〈*J*_*m*_〉} and the floor and ceiling values as, respectively, ⌊〈*J*_*m*_〉⌋ and ⌈〈*J*_*m*_〉⌉. We can estimate the value of *P*(*N*|*D*) to be the following:

(16) $$P\left(N|D\right)=\sum_{m=4}^{n}P\left(m\right)\left[\left(1-\left\{\langle J_{m}\rangle \right\}\right)G\left(m,n-m,\left\lfloor \langle J_{m}\rangle \right\rfloor \right)+\left(\left\{\langle J_{m}\rangle \right\}\right)G\left(m,n-m,\left\lceil \langle J_{m}\rangle \right\rceil \right)\right]$$

For an alternative approach to compute P(*N|D*) that does not involve using mean-field approximations, the following formula can be used, which requires first constructing the distribution *p*(*J*_*m*_):

(17) $$P\left(N|D\right)=\sum_{m=4}^{9}\sum_{J_{m}=0}^{\infty }P\left(m\right)p\left(J_{m}\right)G\left(m,n-m,J_{m}\right)$$

To construct *p*(*J*_*m*_), we first generate all possible local neighborhoods that could surround each *m*-sided central cell, and then compute the expected total number of dividing neighbors for each such neighborhood, which is rounded to a whole number for purposes of substitution into *G*. The distribution *p*(*J*_*m*_) can then be generated by assigning probability mass to each such value of *J*_*m*_ using the multinomial distribution, which gives the chance of observing that particular combination of neighboring cells. Numerical evaluation of equation (17) agrees closely with a Monte Carlo computation using 10^5^ stochastically generated local neighborhoods for each *m*-sided polygonal central cell (Additional file 2: Figure S2). We conclude that the weighted mean-field approximation (equation 16) closely approximates the direct computation (equation 17; see Addititional file 2: Figure S2), and provides an efficient method to compute the distribution *P(N|D)*.

### Predictions of the model

**Figure 3:**
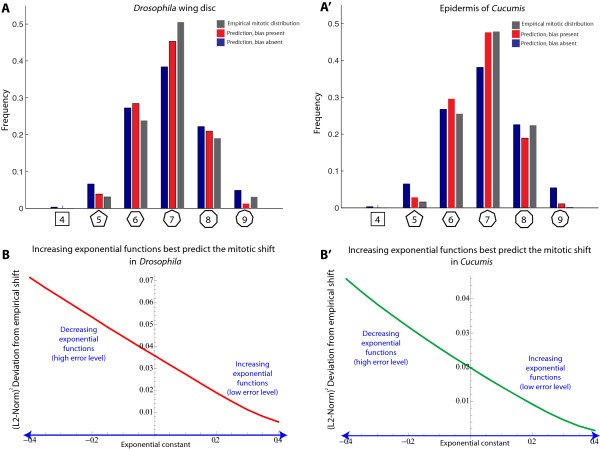
**Computational predictions of the mitotic shift based on a stochastic model of the side-gaining process. (A)** Prediction of the mitotic cell shape distribution for the *Drosophila* wing disc epithelium (*empirical values shown in grey*). In the absence of cleavage plane bias (*blue*), the computational prediction is not quite as accurate as when the bias is included (*red*). Sample sizes for the empirical *Drosophila* mitotic polygonal distribution are given under the heading “Sample sizes for overall and mitotic cell shape distributions” in the Methods section. **(A’)** Prediction of the mitotic cell shape distribution in the epidermis of *Cucumis*. Here, cleavage plane bias similarly improves the accuracy of the prediction. Sample sizes for the empirical *Cucumis* mitotic polygonal distribution are given under the heading “Sample sizes for overall and mitotic cell shape distributions” in the Methods section. **(B)** For the *Drosophila* prediction, a plot of the (l^2^-norm)^2^ deviation from the empirical mitotic cell shape distribution as a function of the relationship between division likelihood and polygon class. Here, division likelihood is assumed to increase exponentially as a function of polygon class. The ordinate (exponential constant) gives the precise form of the exponential. Note that positive values strongly outperform negative values. Hence, a model in which division likelihood increases with polygon class is more consistent with the data than a model in which it decreases or remains the same. **(B’)** For *Cucumis*, the results are nearly identical to those of *Drosophila*.

The predicted form of the mitotic distribution *P(N|D)* depends on the choice of the function *F* (see equation 15). Consistent with the analyses of the first two sections, past studies of *Drosophila* and *Cucumis* monolayer cell sheets demonstrate that the function *F* is monotone increasing [9,19,24]. Specifically, *F* is well fit by an exponential function (based on *Mathematica*’s FindFit function; see references [19,24]). To test whether our modeling framework is able to predict the form of the mitotic cell shape distribution in a proliferating polygonal network, we compared our computational results with empirical data from *Drosophila* and *Cucumis*. In each case, we assumed an exponential form for the function *F*, and searched the parameter space of increasing, decreasing, and flat *F* functions. To measure the distance between the empirical and the predicted form of the mitotic cell shape distribution, we used the square of the l^2^-norm, (l^2^-norm)^2^. Strikingly, for increasing *F* functions, the predicted mitotic distribution *P(N|D)* closely matches the empirical distribution of mitotic cells (Figures 3B-B’; Additional file 2: Figure S2). By contrast, and consistent with the mathematical analysis of the previous sections, flat or decreasing *F* functions failed to match the data closely (Figures 3B-B’; Additional file 2: Figure S2). We find that including cleavage plane bias, *Q(m)*, improves our estimate (compare blue and red bars, Figures 3A-B). These results, which are consistent with more exhaustive modeling approaches, suggest that our analysis is accurate for the case of *Drosophila* and *Cucumis*. Hence, in principle, a simple model of side gaining can account for the mitotic shift in these organisms.

### Passive side gaining drives an increase in polygonal sidedness *in vivo*

**Figure 4:**
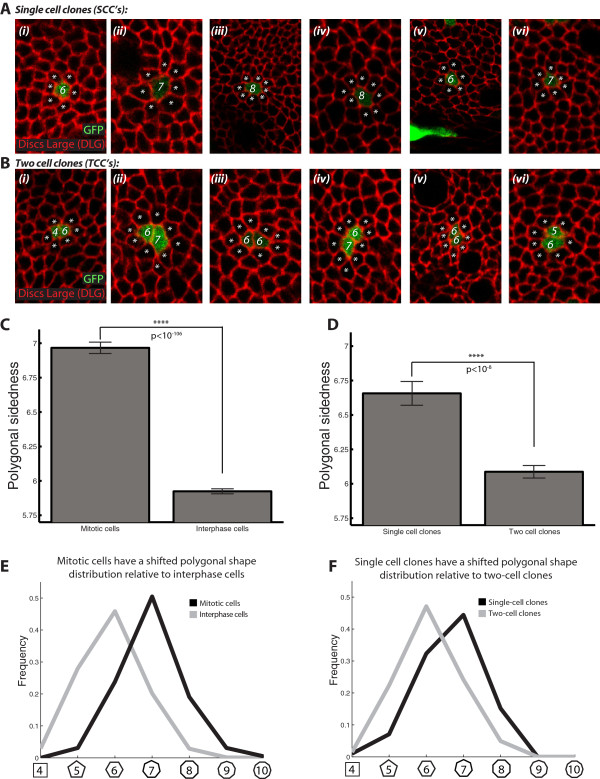
**Statistical summary of the aged cell analysis based on Flp-out clone induction. (A-B)** Induction of Flp-Out clones in the *Drosophila* wing disc epithelium produces cell populations marked with GFP (*green*). Discs Large (DLG; *red*) marks the septate junctions. **(A)** Sub-panels *i-vi* show examples of single cell clones (SCC’s). White stars mark neighboring cells; the labeled cell’s polygonal topology is designated in white. **(B)** Sub-panels *i-vi* show examples of two-cell clones (TCC’s). White stars mark neighboring cells; labeled cells’ polygonal topologies are designated in white. **(C)** The average mitotic cell has approximately seven sides [24], whereas the average non-mitotic cell has approximately six sides [12]. These differences are significant (p < 10^-106^; ttest2 in Matlab). Stars represent statistical significance. Sample sizes for the empirical overall and mitotic distributions are given under the heading “Sample sizes for overall and mitotic cell shape distributions” in the Methods section. **(D)** The average single-cell clone has approximately 6.66 sides, whereas the average two-cell clone has approximately 6.09 sides. These differences are significant (p < 10^-8^; ttest2 in Matlab). Stars represent statistical significance. Sample sizes for the SCC and TCC distributions are given under the heading “Sample sizes for single cell clone (SCC) analysis and two cell clone (TCC) analysis” in the Methods section. **(E)** The mitotic cell shape distribution (*black*) is approximately an integer shift of the overall cell shape distribution (*grey*). **(F)** The single cell clone (SCC) distribution (*black*) is shifted relative to the two-cell clone (TCC) distribution (*grey*). Panels **(E)** and **(F)** display the same data as panels **(C)** and **(D)**, respectively.

Given that the mitotic shift in *Drosophila* (and in other organisms) implies a positive correlation between polygon class and cell cycle state, a natural question is whether this relationship is actually causal. Indeed, it is currently debated whether the mitotic shift merely reflects a time-dependent correlation of two independent processes (cell cycle state and passive side-gaining due to neighboring divisions), or whether polygon class may in some way participate in the active induction of cell division [28,34]. To test whether passive side gaining is sufficient to generate the mitotic shift, we directly measured the polygon class in “aged” *Drosophila* wing disc cells that did not undergo cell division during a twelve-hour time window, and compared this distribution to that observed in cells that underwent a single mitotic event. We stochastically labeled third-instar wing disc epithelial cells with GFP using the FLP-OUT system [37], and permitted the marked cells to grow *in vivo* for 12 hours, the approximate duration of the cell cycle in the wing disc [38-40]. We focused our analysis on two sub-populations of mitotic clones: single cell clones (SCC’s; Figure 4A, *i*-*vi*) and two-cell clones (TCC’s; Figure 4B, *i*-*vi*). SCC’s derive from cells that were GFP labeled during Flp-Out induction, but did not undergo mitosis prior to imaging. To control for the possibility that a subset of the SCC’s derived instead from cell sorting, we discarded SCC’s within two cell diameters of each other (this is a conservative approach, as separated cells are almost never observed at the boundaries of even very large clones in this tissue). We quantified the polygonal topologies of the SCC’s using confocal microscopy and a fluorescently labeled antibody against the septate junction-associated protein Discs Large (Figure 4A-B). Previous studies in diverse organisms have shown both experimentally and mathematically that the average polygonal cell must have exactly six sides in a planar tissue (see Figure 4C; *interphase cells*) [12,20,28,32,41]. By contrast, the population of SCC’s in our sample had on average 6.66 sides 12 hours after clone induction (Figure 4D; *single cell clones*). For comparison, the experimentally measured average polygonal topology of two cell clones was 6.09 (p < 10^-8^ ; t-test2 in Matlab; Figure 4D). We conclude that increased cellular age correlates with increased polygonal sidedness *in vivo*, thus demonstrating that cells experience a net gain in their total number of cell-cell contacts over time.

While SCC analysis is sufficient to detect a net gain in polygonal sidedness, it does not reveal a complete integer shift analogous to the one seen in the mitotic shift (compare Figures 4C and D). To address this discrepancy, we note that a positive correlation exists between division probability and polygonal cell shape (*see previous sections*). SCC analysis is therefore biased topologically, because it only considers labeled cells that have not yet undergone mitosis, which tend to have fewer cell-cell contacts (and were therefore, on average, at an earlier mitotic stage at the time of clone induction). TCC analysis suffers the opposite bias; it only considers labeled cells that have already divided, which show enrichment in cell-cell contacts. Assuming that cell cycle times are roughly asynchronous at the population level, the average pair of daughter cells in a TCC is expected to have divided at the six-hour time point, which is the midpoint of the 12 hour experiment. As the average mitotic cell has seven sides at mitosis, this means that on average, each of the daughter cells is expected to have had 5.5 sides at the six-hour time point. It is therefore notable that the experimentally measured average polygonal topology of two cell clones was 6.09 (Figure 4C). By this reasoning, daughter cells in TCC’s are expected to have gained at least 0.59 sides in a six-hour period. Note this gain in sidedness is approximately ½ of the theoretically expected value of 1 side per cell per cell cycle of 12 hours. Based on these findings, we postulate that side gaining is the primary topological transformation responsible for generating the mitotic shift in the *Drosophila* wing disc.

### Passive side gaining drives an increase in polygonal sidedness *in silico*

**Figure 5:**
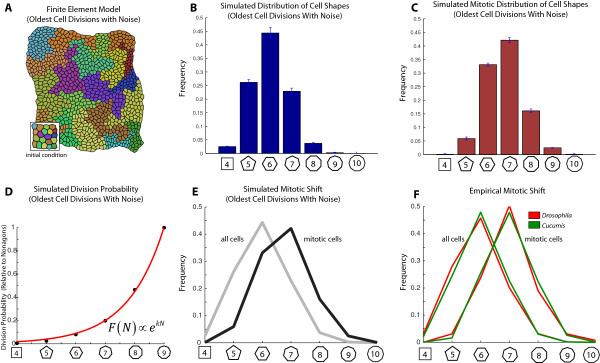
**A finite element model based on oldest cell divisions with noise can recapitulate most features of the mitotic shift. (A)** Initial conditions and model output. Cells are chosen for division as a function of cellular age, with additive noise in the division ordering. The simulated tissue has toroidal boundary conditions, and undergoes at least 1050 mitoses. **(B)** The simulated distribution of cellular shapes. **(C)** The distribution of mitotic cells chosen for division. **(D)** Division likelihood as a function of polygon class, here displayed relative to the likelihood that a nonagon divides (which is taken to be 1). Panel **(D)**, which is computed using Bayes rule, is well fit by an exponential function (the R^2^ coefficient is equal to 0.9989). **(E)** The simulated mitotic shift based on an oldest-cell division mechanism. **(F)** For comparison, the mitotic shift in *Drosophila* and in *Cucumis*. Sample sizes for the empirical overall and mitotic distributions are given under the heading “Sample sizes for overall and mitotic cell shape distributions” in the Methods section.

Taken together, our mathematical and experimental results suggest that the mitotic shift is generated primarily due to the effects of side gaining over the course of the cell cycle, with non-autonomous induction of cell division playing a minimal role, if any. In order to test this hypothesis in a computational framework, we simulated epithelial proliferation (Figure 5A-C) using a finite element model of epithelial morphogenesis, which has been described previously [36]. Cells were chosen for division according to an oldest-cell division rule with additive noise, which simulates a roughly uniform but asynchronous cell cycle schedule. Divisions were implemented according to a longest-axis division rule, consistent with empirical measurements [24]. Consistent with our mathematical analysis from previous sections, this model produces a division likelihood function *F*(*N*) which is well-fit by an exponential function (R^2^ coefficient = 0.9989). Moreover, this approach exhibits a mitotic polygonal shape distribution that is shifted relative to the overall distribution (Figure 5E; compare with Figure 5F). We conclude that passive side-gaining over the course of the cell cycle is sufficient to account for most of the upward shift in polygonal sidedness observed empirically in *Drosophila* and in *Cucumis*.

## Discussion

By combining mathematical and experimental approaches, we have shown that the asynchrony of cell division plays a dominant role in generating the mitotic shift within a proliferating monolayer epithelium. Based on a minimal set of assumptions, for a given dependency between polygon class and division likelihood, we have developed an analytical framework to derive the distribution of mitotic cell shapes. Our mathematical analysis, experimental results, and finite element simulations together suggest that the mitotic shift is a topological phenomenon that is primarily a consequence of the correlation between autonomous cell cycle progression and non-autonomous side gaining. Some systems may rely on additional, redundant, shape-dependent cell division induction mechanisms [28,34]. However, particularly in light of the fact that the mitotic shift is manifest in independently evolved forms of multicellular life [33], the most parsimonious conclusion is that such mechanisms are not required even if they cannot be completely ruled out.

To test for a subtle role of mechanical stress in generating the mitotic shift in the *Drosophila* wing disc, advances in live imaging may eventually permit precise tracking of both cellular age and of cellular geometry over time [24,42-44]. Even if a small geometric influence could be detected, the divergent genetics and divergent mechanics of plant epidermis and animal epithelia make it unlikely that such a mechanism would be conserved across kingdoms. Hence, such a hypothesized influence would most likely be a feature of a particular tissue, not a general explanation for the shift. The framework developed here places strong quantitative limits on the possible contribution of cellular geometry (or correlative mechanical stress), while simultaneously demonstrating the dominance of the division process.

Looking forward, our results lead to several questions for future analysis. Perhaps the most critical would be to ask how the frequency of cell-cell rearrangement impacts the dynamics considered here, especially if there were topological biases in the rates of different cell-cell movements. Previous studies in *Drosophila* wing discs have reported variable rates of neighbor exchange events, ranging from negligibly low [12] to high [45]. At one unlikely extreme, a very high degree of undirected neighbor exchange events could essentially erase the mitotic shift and drive the tissue towards a hexagonal topology. This is itself an argument against the existence of large-scale neighbor exchanges in the developing wing imaginal disc. At the other extreme, a low degree of neighbor exchange events, particularly if they favored particular topological transformations, could produce more subtle perturbations of the mitotic shift or the global distribution of cell shapes. Since cell movements within epithelia are likely to play a key role in different aspects of tissue morphogenesis, understanding their implications for both the overall topology and the mitotic shift could be a key avenue for future studies.

## Methods

### Numerical computations

Numerical computations were performed in *Mathematica* 8.0 (Wolfram Research, Inc.). For parameter fitting, *Mathematica*’s FindFit function was used. A full description of the methods for implementing finite-element based simulations of epithelial proliferation (see Figure 5) and topological simulations of epithelial proliferation (see Additional file 1: Figure S1) can be found elsewhere [24]. Simulation results presented in Figure 5 are based on runs that were replicated in triplicate, with each run containing at least 1050 cell divisions. Simulation results presented in Additional file 1: Figure S1 were also replicated in triplicate, with each run containing at least 80,000 cell divisions.

### Fly strains

To visualize the septate junctions (Figure 1), we used a *neuroglian-gfp* exon trap line, which was described in a previous study (*nrg-gfp*; [46]).GFP-expressing clones (Figure 4) were induced in flies of the following genotype:

yw hs-flp^122^; Actin5c> > Gal4,UAS-GFP/+ with a 30-minute heat shock at 37C followed by a 12-hour recovery period prior to dissection.

### Immunohistochemistry

Wing discs expressing marked clones (Figure 4) were stained with mouse anti-discs large (1:1000 dilution, DSHB) to mark the septate junctions.

### Wing Disc sample preparation and Imaging

Wing discs were dissected from wandering 3rd instar larvae in Ringers’ solution, fixed in 4% paraformaldehyde in PBS, and mounted in 70% glycerol/PBS. Discs were imaged on a Leica SP5 with a 63× glycerol objective.

### Image processing procedures

Single cell clones (SCC’s) and two cell clones (TCC’s) were imaged in multiple focal planes, and were displayed as two-color image stacks (one color for the Flp-Out GFP, and one color for anti-discs large or neuroglian-GFP) in Leica’s LAS AF imaging software for the SP5 confocal microscope. Analysis was performed by hand; cells having ambiguous polygonal topology were not counted. To control for the possibility of cell sorting, SCC’s and/or TCC’s were not considered for analysis unless they were separated by at least two cell diameters within the tissue. In order to control for boundary effects, cells located on tissue folds close to the anterior-posterior (AP) or dorsal-ventral (DV) compartment boundaries were not counted. To prevent mis-identification of SCC or TCC clones, we did not consider cells for scoring if the source of the GFP signal was ambiguous (for example, if the GFP source overlapped with another bright clone in a different focal plane).For display (non-analytical) purposes, Figure 1D shows mitotic cells that have been first inverted, and then subjected to a brightness threshold cutoff in Adobe Photoshop.

### Sample sizes for single cell clone (SCC) analysis and two cell clone (TCC) analysis

Samples sizes used to compute each polygonal cell shape’s respective frequency for single cell clones (SCC’s) and two cell clones (TCC’s) are as follows: Single cell clones, (4, 1; 5, 7; 6, 32; 7, 44; 8, 15; 9, 0; 10, 0). Two cell clones, (4, 5; 5, 77; 6, 162; 7, 83; 8, 17; 9, 0; 10, 0).

### Sample sizes and polygonal counts by organism

Sample sizes used to compute each polygonal cell shape’s respective frequency in *Drosophila *[12], *Xenopus *[12], *Hydra *[12], and *Cucumis *[9], have been previously described. For reference, these are as follows: *Drosophila*, (4, 64; 5, 606; 6, 993; 7, 437; 8, 69; 9,3). *Xenopus*, (3, 2; 4, 40; 5, 305; 6, 451; 7, 191; 8, 52; 9, 8; 10, 2), *Hydra,* (4, 16; 5, 159; 6, 278; 7, 125; 8, 23; 9, 1). *Cucumis*, (4, 20; 5, 251; 6, 474; 7, 224; 8, 30; 9, 1). Aggregate sample sizes and polygonal frequencies for *Allium*, *Euonymus*, *Dryopteris*, and *Anacharis* have been previously described [32]. For reference, these are as follows: *Allium* (*n* = 500 cells), (4, 0.040; 5, 0.302; 6, 0.412; 7, 0.181; 8, 0.047; 9, 0.015). *Euonymous* (*n* = 200 cells), (4, 0.030; 5, 0.290; 6, 0.400; 7, 0.220; 8, 0.066; 9, 0). *Dryopteris* (*n* = 200 cells), (4, 0.040; 5, 0.260; 6, 0.425; 7, 0.205; 8, 0.050; 9, 0.020). *Anacharis* (leaf, abaxial, *n* = 200 cells), (4, 0.025; 5, 0.240; 6, 0.595; 7, 0.100; 8, 0.035; 9, 0). *Anacharis* (leaf, adaxial, *n* = 200 cells), (4, 0.035; 5, 0.255; 6, 0.570; 7, 0.130; 8, 0.010; 9, 0). *Anacharis* (bud, *n* = 200 cells), (4, 0.055; 5, 0.295; 6, 0.450; 7, 0.200; 8, 0; 9, 0).

### Sample sizes for overall and mitotic cell shape distributions

Sample sizes used to compute each polygonal cell shape’s respective frequency for resting and mitotic cells, respectively, in both *Drosophila *[12,24] and *Cucumis *[9], have been described previously. For reference, these are as follows: *Drosophila* (overall cell shape distribution), (4, 64; 5, 606; 6, 993; 7, 437; 8, 69; 9,3). *Drosophila* (mitotic cell shape distribution), (4, 0; 5, 13; 6, 100; 7, 212; 8, 80; 9, 13; 10, 3). *Cucumis* (overall cell shape distribution), (4, 20; 5, 251; 6, 474; 7, 224; 8, 30; 9, 1). *Cucumis* (mitotic cell shape distribution), (4, 0; 5, 16; 6, 255; 7, 478; 8, 224; 9, 26; 10,1).

## Competing interest

The authors declare that they have no competing interests.

## Authors’ contributions

WTG, BYR, EJM, and JHV performed research. WTG, BYR, MCG designed research. GWB, RN, and MCG supervised research. WTG, BYR, and MCG wrote the paper. WTG and BYR contributed equally to this work. All authors read and approved the final manuscript.

## Supplementary Material

Additional file 1: Figure S1 Computational support to show that the mitotic shift is absent when the probability of mitotic entry is uncorrelated with polygon class. (A-B) Initial conditions and model output, respectively. (C) Cell division is simulated as a two-step process. First, a new tri-cellular junction is inserted into one of the dividing cell’s edges, with probability proportional to specified weights, which are either uniform (all edges shared with neighboring polygons have equal weight) or exponentially biased (edges shared with neighboring polygons have exponentially smaller weight as a function of the number of edges of that polygon). Here, the exponential parameter is 2.7 (i.e., pentagons have 2.7 times as much weight as hexagons). The second step of the algorithm decides the edge into which a second new tri-cellular junction will be inserted by sampling from a division kernel matrix (see [24] for details). The final step of the algorithm is to connect the two new tri-cellular junctions to form the cleavage plane. (D**-**F) When the division kernel matrix is maximally symmetric (octagons divide into pairs of hexagons, etc.), and no cleavage plane bias is present, a random division timing model produces no mitotic shift. Colors denote separate runs; error bars refer to the standard deviation in polygon frequency. Simulations proceed until the population reaches at least 80,000 cells. A lack of a mitotic shift is also found in cases when the division kernel matrix is symmetric but cleavage plane bias is present (G**-**I). The same result is also found in the absence (J**-**L) or presence (M**-**O) of such bias when the division kernel is binomially distributed. These data are consistent with the interpretation that the mitotic shift is absent when divisions are simulated as a Poisson process in which every cell is equally likely to divide per time step. Click here for file

Additional file 2: Figure S2 An overlay of three different approaches for computing the mitotic cell shape distribution *P(N|D)* in the *Drosophila* wing disc. (A) For each approach, the function *F* is assumed to be exponential. Results are compared in terms of the l-2 norm squared, as a function of the exponential constant in *F*. For the Monte Carlo approximation **(*red*)**, we have computed the expected number of neighbor cell divisions for a stochastically generated set of 10^5^ local neighborhoods for each class of central cell polygon. Using these neighborhoods, we numerically constructed an approximate distribution of *Jm* values for each *m*. The total number of expected neighbor cell divisions is rounded to a whole number for each local neighborhood, which is a constraint imposed by the *G* function. For the exact numerical computation **(*black****;* see equation (17)), for each class of central cell polygon, we computed the expected number of neighbor cell divisions for every possible combination of neighbors, and used it to construct the distribution of *Jm* values based on the probability of observing each neighborhood type, as given by the multinomial distribution. For each of the possible neighborhood types, as required by the *G* function, we rounded the total number of expected neighbor cell divisions to a whole number. For the mean field computation using linear weights ***(blue****;* see equation (16)), an average of two evaluations of the *G* function are used (see equation 16), one using the truncated (floor) value for the mean-field estimate of *Jm*, and the other using the ceiling (next greatest integer) for the mean field estimate of *Jm*. All three methods give similar results, which strongly suggests that equation (16) is a good approximation for the exact computation (equation 17). Click here for file
