# Supplementary material for: On the origins of the mitotic shift in proliferating cell layers
Source: Theor Biol Med Model. 2014 May 27;11:26. doi: 10.1186/1742-4682-11-26 (PMC4048254; doi:10.1186/1742-4682-11-26)

Figure S1:

Topological simulations confirm that the mitotic shift is absent when mitoses occur with random timing.

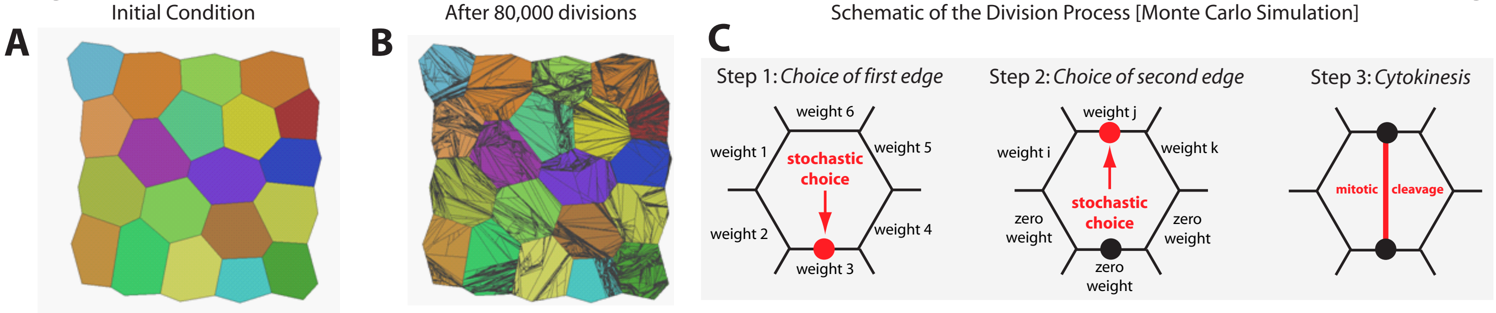

Maximally symmetric divisions [cleavage planes are unbiased with respect to neighor cell geometry]

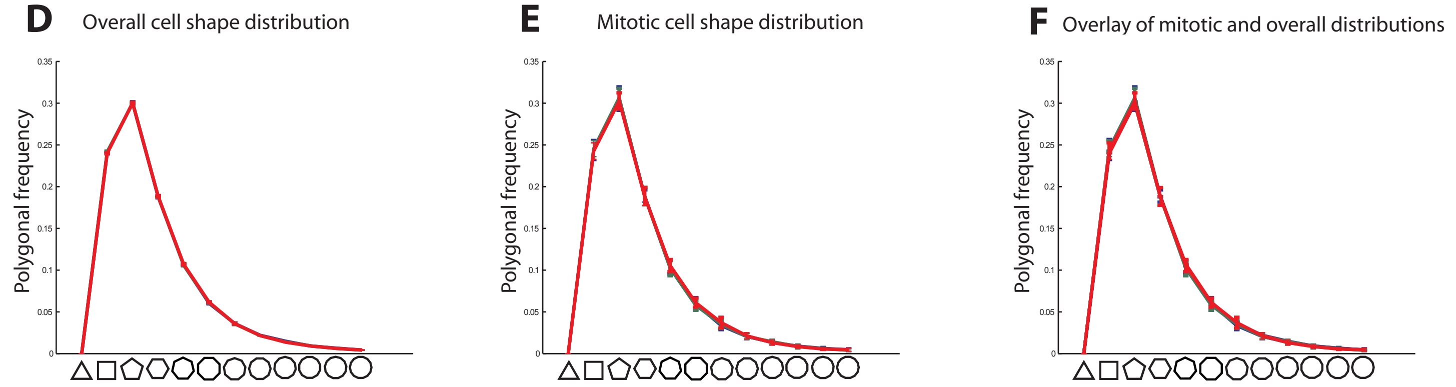

Maximally symmetric divisions [cleavage planes are biased with respect to neighbor cell geometry]

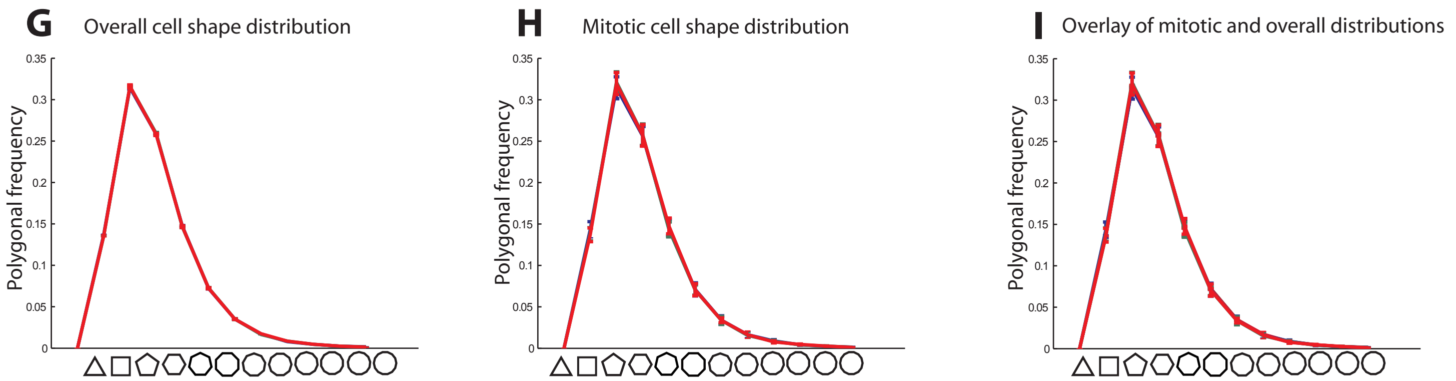

Binomial distribution of mother cell junctions to daughters [CP's are unbiased with respect to neighbor cell geometry]

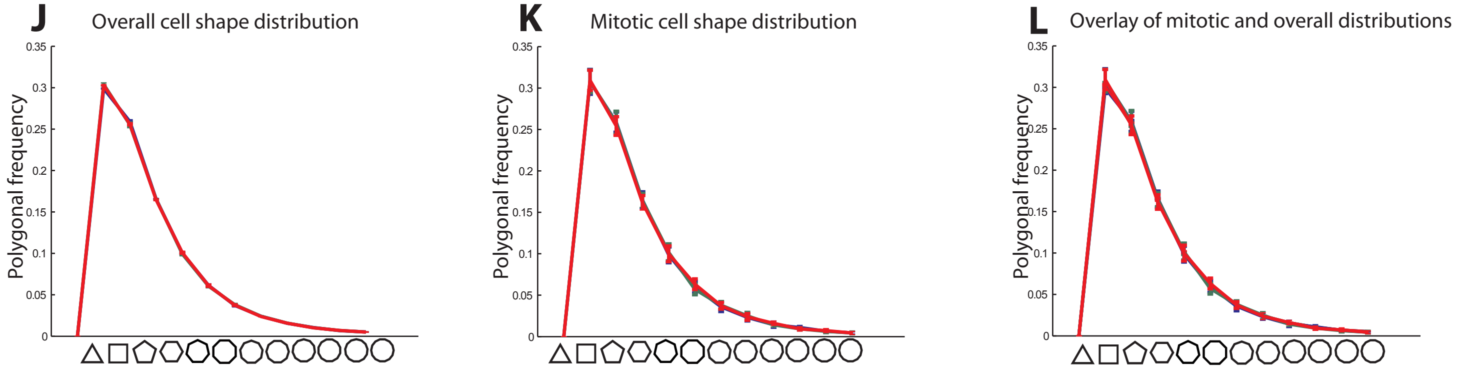

Binomial distribution of mother cell junctions to daughters [CP's are biased with respect to neighbor cell geometry]

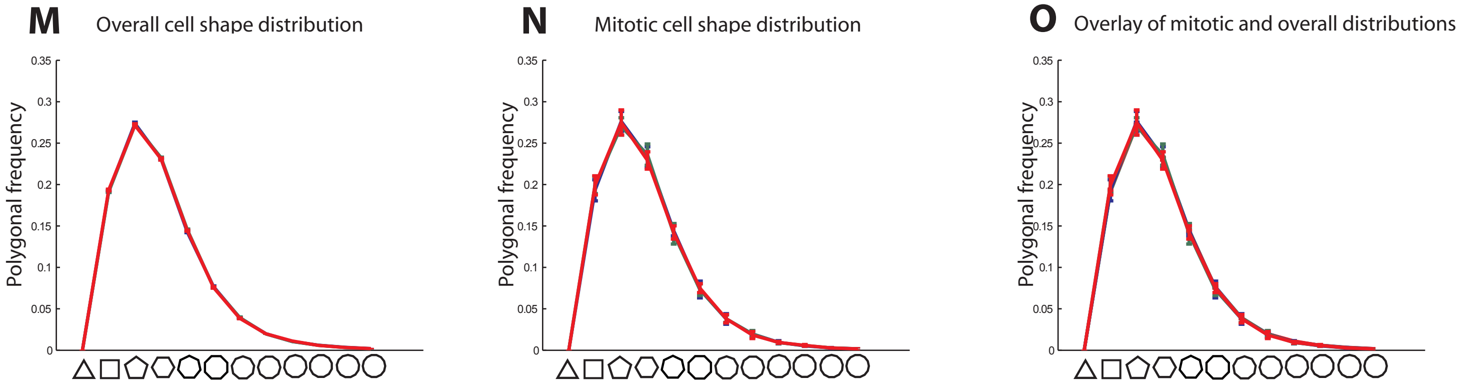

Supplement: Additional file 1: Figure S1 — Computational support to show that the mitotic shift is absent when the probability of mitotic entry is uncorrelated with polygon class. (A-B) Initial conditions and model output, respectively. (C) Cell division is simulated as a two-step process. First, a new tri-cellular junction is inserted into one of the dividing cell’s edges, with probability proportional to specified weights, which are either uniform (all edges shared with neighboring polygons have equal weight) or exponentially biased (edges shared with neighboring polygons have exponentially smaller weight as a function of the number of edges of that polygon). Here, the exponential parameter is 2.7 (i.e., pentagons have 2.7 times as much weight as hexagons). The second step of the algorithm decides the edge into which a second new tri-cellular junction will be inserted by sampling from a division kernel matrix (see [24] for details). The final step of the algorithm is to connect the two new tri-cellular junctions to form the cleavage plane. (D-F) When the division kernel matrix is maximally symmetric (octagons divide into pairs of hexagons, etc.), and no cleavage plane bias is present, a random division timing model produces no mitotic shift. Colors denote separate runs; error bars refer to the standard deviation in polygon frequency. Simulations proceed until the population reaches at least 80,000 cells. A lack of a mitotic shift is also found in cases when the division kernel matrix is symmetric but cleavage plane bias is present (G-I). The same result is also found in the absence (J-L) or presence (M-O) of such bias when the division kernel is binomially distributed. These data are consistent with the interpretation that the mitotic shift is absent when divisions are simulated as a Poisson process in which every cell is equally likely to divide per time step. [file 1742-4682-11-26-S1.pdf]
