# Supplementary figures and images for: On the origins of the mitotic shift in proliferating cell layers
Source: Theor Biol Med Model. 2014 May 27;11:26. doi: 10.1186/1742-4682-11-26 (PMC4048254; doi:10.1186/1742-4682-11-26)

**Figure S2**

**A** Fit error for estimating  $P(N|D)$  in the side-gaining model (*Drosophila* wing disc)

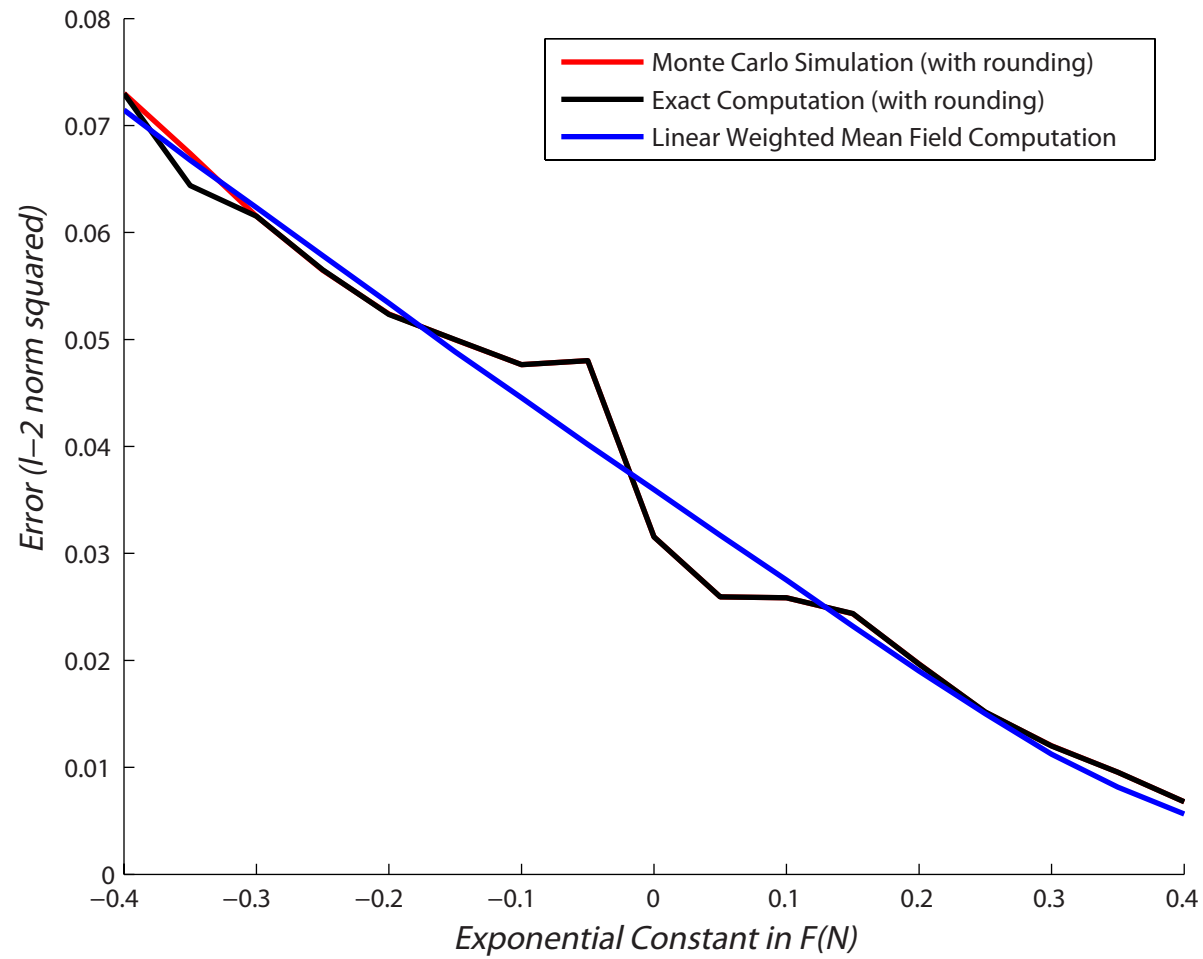

Supplement: Additional file 2: Figure S2 — An overlay of three different approaches for computing the mitotic cell shape distribution P(N|D) in the Drosophila wing disc. (A) For each approach, the function F is assumed to be exponential. Results are compared in terms of the l-2 norm squared, as a function of the exponential constant in F. For the Monte Carlo approximation (red), we have computed the expected number of neighbor cell divisions for a stochastically generated set of 105 local neighborhoods for each class of central cell polygon. Using these neighborhoods, we numerically constructed an approximate distribution of Jm values for each m. The total number of expected neighbor cell divisions is rounded to a whole number for each local neighborhood, which is a constraint imposed by the G function. For the exact numerical computation (black; see equation (17)), for each class of central cell polygon, we computed the expected number of neighbor cell divisions for every possible combination of neighbors, and used it to construct the distribution of Jm values based on the probability of observing each neighborhood type, as given by the multinomial distribution. For each of the possible neighborhood types, as required by the G function, we rounded the total number of expected neighbor cell divisions to a whole number. For the mean field computation using linear weights (blue; see equation (16)), an average of two evaluations of the G function are used (see equation 16), one using the truncated (floor) value for the mean-field estimate of Jm, and the other using the ceiling (next greatest integer) for the mean field estimate of Jm. All three methods give similar results, which strongly suggests that equation (16) is a good approximation for the exact computation (equation 17). [file 1742-4682-11-26-S2.pdf]
